# Conductomeric Evaluation of the Release Kinetics of Active Substances from Pharmaceutical Preparations Containing Iron Ions

**DOI:** 10.3390/ma12050730

**Published:** 2019-03-03

**Authors:** Anna Lisik, Witold Musiał

**Affiliations:** Department of Physical Chemistry, Pharmaceutical Faculty, Wroclaw Medical University, Borowska 211, 50-556 Wroclaw, Poland; anna.lisik@student.umed.wroc.pl

**Keywords:** electric conductivity, release kinetics, drug release models, drug dissolution, controlled drug release, electrochemistry, metal complexes

## Abstract

The aim of this study was to verify the effect of the formulation on the release kinetics of active substances from preparations containing iron ions using in-line conductivity measurements. A simple, fast method was developed and may be applied for detailed evaluation of some kinetics factors obtained from the release data. Four different equations were used: zero-order equation, first-order equation, models: Korsmeyer–Peppas and Hixson–Crowell. Values of the determined half-time release for zero and first-order kinetic models ranged from 11.56 to 89.97 min. In the case of analysis according to these typical models, the values of the square root of the correlation coefficients were included between 0.9916 and 0.9995. The results transformed for the Hixson–Crowell model as constant release Ks, ranged between 0.0160 and 0.0437. The values of the respective calculated squares of the correlation coefficient ranged from 0.9933 to 0.9959. The determined release rate constants according to the Korsmeyer–Peppas model were between 0.0023 and 0.1630. The coefficients ‘n’ of the Korsmeyer–Peppas equation did not exceed 1.2 with the corresponding r^2^ values 0.9408–0.9960. Obtained results confirmed that the method is applicable for evaluation of selected drug compositions containing iron ions.

## 1. Introduction

The variety of analytical methods is applied to obtain detailed insight into the qualitative and quantitative aspects of drug release from the solid formulations. Numerous authors proposed high performance liquid chromatography (HPLC) in its different versions, e.g.: HPLC with ultra violet (UV) [1] or diode-array detector (DAD) detection [2], nowadays frequently combined [3]. The wide possibilities in the detection of released bioactives include: evaporative light scattering detector (ELSD) [4], refractive index detector [5], contactless conductivity detector [6], the reversed phase HPLC [7], ultra HPLC [8], as well as gas chromatography (GC) [9] and liquid chromatography–mass spectrometry (LCMS) [10], are also applied in the field of the assessment of the bioctives release from the formulation. Anyway, the most applied methods include UV–Vis [11,12] and atomic absorption spectrometry (AAS) [13], as useful and cost-effective methods. The above-mentioned analytical methods often enable frequent recording of increasing concentrations of the analyte in dissolution medium. In our department, we evaluated a new method designed for in-line assessment of release of bioactives from solid drug forms. The method was partially validated, and thus may be applied for detailed evaluation of some kinetics factors obtained from release data [14]. The data obtained by continuous multiplying measurements result in detailed course of release plot and may be used for kinetic model calculations. In most cases, kinetic models are used when new drug formulation is evaluated to confirm the effectiveness of drug release. Selection of the appropriate mathematical model when developing new pharmaceutical products or elucidation of the drug release mechanisms strongly depends on the precision and accuracy of the model. In selected cases, the use of a simple mathematical model is sufficient. However, the release data, gathered as release profiles, may be used for preliminary reflection of bioavailability of the drug in specific drug form. In this research, four equations were used: zero-order equation, first-order equation, Korsmeyer–Peppas, and Hixson–Crowell, as presented in Table 1.

In the zero-order model the constant amount of active pharmaceutical substance (API) is released per time unit, whereas in the first-order model there is a constant proportion of drug released per time unit. However, the first-order process depends on the concentration, and the zero-order kinetics is actually independent of concentration. Both models are valuable when the pharmacokinetic study is concentrated on differentiation of potential preparations, which may be applied as controlled release products. The ideal situation may reflect the zero-order process, when the initial and actual drug concentration in the formulation does not influence the release rate. More detailed models may enable approximation of the mechanism which are influencing the drug amounts released into the dissolution medium. In 1983 Korsmeyer, Gurny, Doelker, Buri, and Peppas developed a model for description of drug release from polymeric systems. The mechanism of drug release may be detailed by adoption of the initial 60% of the semi-empirical model, and is known as the Korsmeyer–Peppas model. The value ‘n’ in the model reflects the possible release mechanisms of drug according to Table 2 [15,16,17,18,19,20,21].

The calculation of the value of exponent ‘n’ may be performed on the plot part where: M_t_/M_∞_ < 0.6. In 1931 Hixson and Crowell recognized that the rate of dissolution depends on the surface contacting with the applied solvent. The increase in the surface area results in faster dissolution. This model fits well to the drug release from the tablets, due to the respective changes in surface area and diameter [15,16,17,18,19,20,21].

The purpose of the following study was to adjust the selected kinetic models, to the results obtained from the conductometric measurements of the release of iron ions from three drugs available on the European pharmaceutical market. The previously developed new analytical method was partially validated and applied in the present study [14].

## 2. Materials and Methods 

### 2.1. Materials

Three various preparations, obtained from the market, containing iron ions were used in the study. Composition FEP1 contained 100 mg of iron as ferrous sulfate and 60 mg of ascorbic acid, in the form of tablet with prolonged release of bioactives. Composition FEP2 included 105 mg of iron ions as ferrous sulfate, and 0.35 mg of folic acid. FEN3 included iron as iron (II) gluconate corresponding to 23.2 mg of iron, tablets form. Deionized water from dedicated Hydrolab HLP20UV device (Hydrolab Sp. z o.o. Sp.K., Straszyn, Poland) was used in all the conductivity assays [14].

### 2.2. Dissolution Test

The dissolution tester Erweka DT 700 (Erweka GmbH, Heusenstamm, Germany) with paddle at 75 rpm was used, for the determination of the formulation dissolution, according to pharmacopoeial standards. Evaluated tablets were placed in the bottom of dissolution vessels and dissolution test according to the Ph. Eur. was performed in this experiment [22]. Purified water of 37 °C as a dissolution medium, in a volume of 900 mL was used during entire experiment [14].

### 2.3. Electric Conductivity Method Conditions

Sample solution preparation: sample solution preparations were obtained from the dissolution studies performed via dissolution paddle. Conductivity probe (Elmetron conductivity meter CC-505 with conductivity probe EC-70, Elmetron, Zabrze, Poland) was placed in the middle of dissolution vessel and the result was read directly from the conductivity meter in µs·cm^−1^. Measurement using conductivity meter was performed in every 5 min until the equilibrium state was reached for each composition: 180 min for composition FEP1 and FEP2 and 50 min for composition FEN3. The study was performed in six vessels on every composition, and the calibration curves for conductivity evaluation were prepared from the powdered solid formulations. The electric conductivity method has been developed and partially validated for all three drug compositions as per ICH guidelines [23], and presented in former paper [14].

### 2.4. Calculations

The basic calculations were performed in the Excel Worksheet enhanced by trial Analytic Solver Basic by FrontlineSolvers. Four basic kinetic models were applied for the calculations: zero-order, first-order, Korsmeyer–Peppas, and Hixson–Crowell. Determination coefficients for obtained rates, and specific factors were calculated as squares of respective correlation coefficients. The data for the zero-order, first-order and Hixson–Crowell kinetics were evaluated “duo modo”: firstly, the basic data were calculated according to the specified equation, as presented in Table 1, numbers 1–3. The fitting segments, represented by straight lines, were used as a basis for sketching the respective kinetics.

## 3. Results

**Figure 1:**
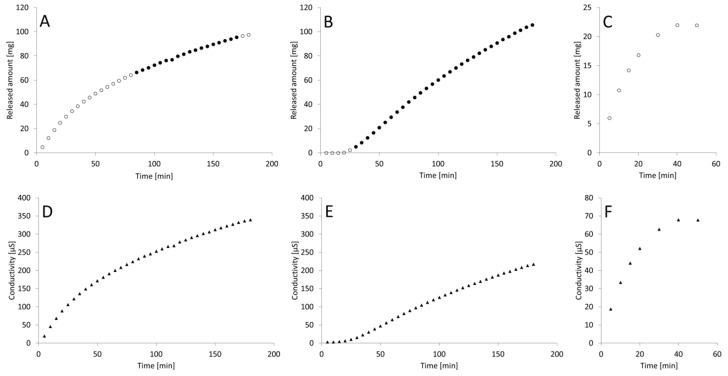
The process of API release from tested preparations containing ferric ions, determined by the conductometric method. A–C: release profiles of preparations FEP1 (**A**), FEP2 (**B**), FEN3 (**C**), ○—experimental data; ●—experimental data applied for the evaluation of approximated zero-order kinetics. D–C: raw conductivity results (▲) for FEP1 (**D**), FEP2 (**E**), FEN3 (**F**).

The process of drug substance release from the tested tablets is illustrated in Figure 1. The preparation A (FEP1) released almost 100% of bioactive within about 180 min, while during the first 100 min the process is close to the exponential function. In the case of preparation B (FEP2), in the first 30 min no release of the drug substance was observed; after this period the progress of release was curvilinear, although the curve was rather flattened and slightly deviates from the course of the straight line. As in the case of preparation A, almost all of the active substance proceeds into the dissolution medium after about 180 min. The release of the active substance from preparation C (FEN3) was definitely different. The active substance from formulation C released rapidly, reaching a plateau at 40 min, which corresponded to about 100% of released analyte. The original, non-transformed data, obtained in the course of in-line conductivity measurements, are presented in Figure 1D–F.

Table 3 summarizes the calculated parameters of kinetic equations describing the process of API release from individual formulations: FEP1, FEP2, FEN3. In pursuance of the kinetic calculations carried out according to the 0 order and 1 order kinetic model, the process of API release from FEP1 was characterized by two distinct phases with specific constants, whereas the release from FEP2 and FEN3 could be clearly presented as a one-step process. Values of the determined half-time release for these kinetic models ranged from 11.56 to 89.97 min. Table 1 also presents the values of the square root of the correlation coefficient, informing about the adjustment of the applied model to the observed progress of the examined process. In the case of analysis according to these typical models the values were included between 0.9916 and 0.9995. The results of observations collected in subsequent columns were transformed for the Hixson–Crowell model as constant release Ks, ranged between 0.0160 and 0.0437 with the highest constant for FEN3. The values of the respective calculated squares of the correlation coefficient ranged from 0.9933 to 0.9959. The determined release rate constants according to the Korsmeyer–Peppas model were from very low 0.0023 for FEP2 to 0.1630 for FEN3. The coefficients ‘n’ of the Korsmeyer–Peppas equation did not exceed 1.2, with the corresponding r^2^ values from 0.9408 to 0.9960.

## 4. Discussion

**Figure 2:**
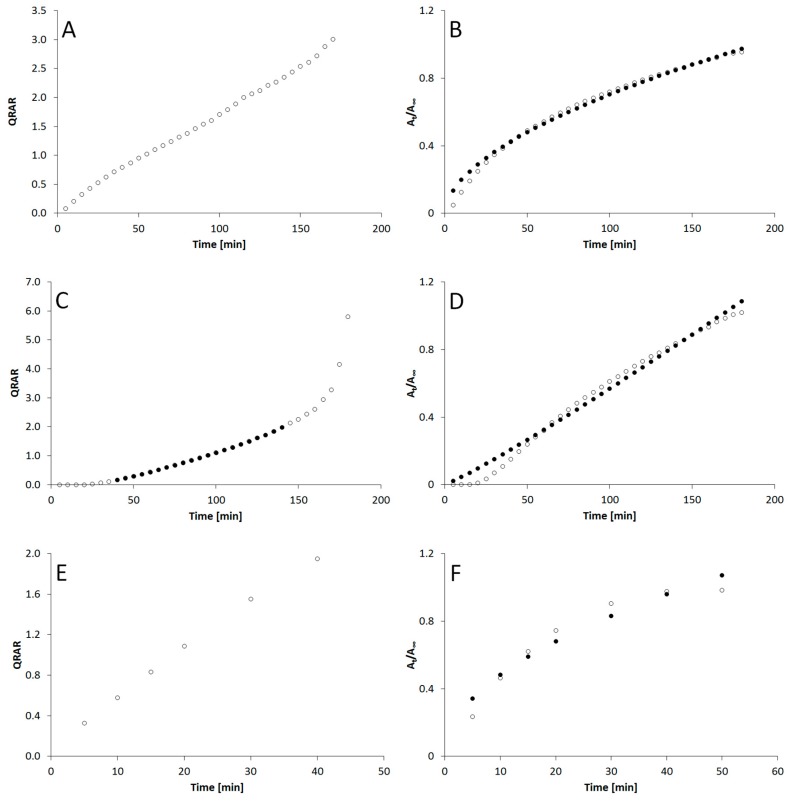
The Hixson–Crowell (**A**,**C**,**E**) and Korsmeyer–Peppas (**B**,**D**,**F**) kinetics, applied to evaluated preparations: FEP1 (**A**,**B**), FEP2 (**C**,**D**) and FEN3 (**E**,**F**) respectively. The filled circles on panel C represent the partial fitting of the data to the Hixson–Crowell model in the case of FEP1. The filled circles on the panels B, D, F represent the data calculated for the Korsmeyer–Peppas model according to the best fitted section of the graph in the SOLVER application, whereas the empty circles on these panels represent the surrogate data transformed according to the model. The empty circles on the axis x on panel D should not be included for the consideration of Korsmeyer–Peppas model for FEP2. QRAR—cubic root of the amount of released drug, A_t_/A_∞_ the ratio of the amount of drug released at time t to the total released amount of drug.

Figure 2 presents the course of kinetic curves describing the process of API release for individual preparations (FEP1, FEP2 and FEN3) according to the proposed Hixson–Crowell and Korsmeyer–Peppas models.

**Figure 3:**
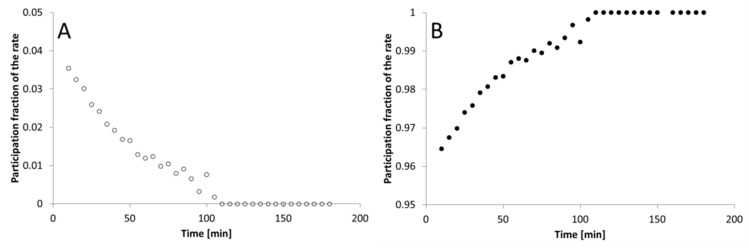
Decrease of k₁ participation in the kinetics process (**A**), and increase of k₀ participation in the kinetics process (**B**) in the total release process depending on time for the preparation FEP1.

Based on dependence of the substance amount released in the time (Figure 1A) it can be concluded that from the preparation A the active substance is released in a mixed manner: (a) calculations performed with the Solver program allowed to determine that the API release from this preparation in the first stage i.e. up to about 100 min of the process is characterized by the advantage of the first-order kinetics. However, after this period, the process is first-order in the next 80 min. Figure 3A shows the change of participation of first-order kinetics in the whole process and zero-order in the overall process depending on time (Figure 3B).

The diversity of this process can be summarized by the equation:

∆C = Ak_1_ × C_0_ ∆t + Bk_0_ × ∆t (1)

where: A—fraction of the 1st order kinetics at a given moment of the release process, B—fraction of the 0th order kinetics at a given moment of the release process, t—time, C concentration at a specific moment, C_0_—initial concentration, k_0_—zero-order rate constant, k_1_—first-order rate constant.

The observed process may reflect tablet technological design which is a compact tablet covered by thin polymer film and contains both the initiating and the maintenance dose. The release process graph for the Hixson–Crowell model (Figure 2A) does not confirm the complete compatibility of the release kinetics with the surface drug loss, although relatively high r^2^ coefficient values were obtained for this model (Table 3). According to the value 0.574 of coefficient n for the Korsmeyer–Peppas model preparation A releases the active substance both as a result of diffusion and as a result of erosion. Reference values for so-called anomalous transport are within the limits between 0.5 and 1.0. The release process according to this model is given in Figure 2B.

In the case of preparation B independently of the amount of active substance in the donor compartment, the release process was clearly observed which can be related to the limiting effect of a thick water-insoluble coating. Also the lag-time clearly observed in the case of preparation B indicates the important role of the coating in the drug substance release in this case compared to preparation A, the graph prepared for the Hixson–Crowell model presented as a straight line in the limited extent which indicates the effect of the formulation surface loss on the release rate at the time (Figure 2C); It should be clearly stated that the process consistent with this model was observed only in a limited period of time between 40 and 140 min. Confirmation of the surface loss as a release controlling factor is here a high value of the ‘n’ coefficient in the Korsmeyer–Peppas model exceeding the value of 1.0 called the super case II transport (Table 3, Figure 2D) [24].

The active substance of preparation C was released according to the first-order kinetics. The release rate decreased over the time, corresponding to the situation with unmodified but long-term releasing tablets. Formulation C was characterized by a strongly linear graph plot for the Hixson–Crowell model almost in the entire period of time studied as shown in Figure 2E. This suggests an outstanding conection between the loss of product surface and the amount of the therapeutic substance released. However, this fact is not directly confirmed in the process flow compared to the Korsmeyer–Peppas model; the value of the corresponding factor ‘n’ for the preparation C does not exceed 0.5, ie the characteristic value for the diffusion described by the Fick equation. The respective release process is illustrated in Figure 2F.

Future evaluation of the conductometric assays should involve analysis of subsequent preparations containing iron ions to wider the application field of the above-presented method. Development of a database with the results of conductometric assays for excipients may significantly facilitate the pharmaceutical analytics with the hereby presented method for numerous solid forms containing other ions. The limitations of the method include variability of the conductivity of aqueous solvent, and influence of impurities on the conductivity of the samples.

## 5. Conclusions

Application of conductometric measurements enables comparative assessment of the drug release kinetics from preparations containing iron ions. The proposed analytical method is applicable for the evaluation of the release process in accordance with complex kinetic models including zero-order and 1st kinetics, as well as KP and HC models. The revealed variability of kinetics via conductometric method enables differentiation of pharmaceutical preparations according to the drug release mode on the molecular level.

## Figures and Tables

**Table 1:** Selected kinetic models applied for the evaluation of release kinetics.

Kinetic Model	Equation	Source
zero-order	$C_{t}=C_{0}+k_{0}t$	[15,16,17]
first-order	$\log C_{0}-\log C_{t}=\frac{k_{1}t}{2.303}$	[15,16,17]
Hixson–Crowell	$W_{0}^{\frac{1}{3}}-W_{t}^{\frac{1}{3}}=\kappa t$	[15,16,17]
Korsmeyer–Peppas	$F=\left(\frac{M_{t}}{M}\right)=K_{m}\cdot t^{n}$	[15,16,17]

k_0_—zero-order constant; k_1_—first-order constant; C_0_—an initial concentration of released drug; C_t_—the concentration of drug released in the time; W_0_—the initial mass of drug in the pharmaceutical dosage form; W_t_—the remaining mass of drug in the pharmaceutical dosage form at time t; κ—the constant value combine the surface and volume relation; F—fraction of drug released at time t; M_t_—the mass of drug released at time t; M—the total mass of drug in dosage form; K_m_—kinetic constant; n—diffusion or release exponent; t—time in hours.

**Table 2:** The variability of release exponent (n) and the respective release mechanisms.

n	Drug Transport Mechanism	Rate as a Function of Time (t) Transformation
0.5	Fickian diffusion	t^−0.5^
0.45 < n = 0.89	Non-Fickian transport	t^n−1^
0.89	Case II transport	t
>0.89	Super case II transport	t^n−1^

**Table 3:** Basic kinetic parameters of the process of API kinetics release from the tested preparations: FEP1, FEP2, and FEN3.

Composition	k_1_ (1/min)	k_0_ (mg/min)	t_0.5_ (min)	r^2^	K_S_	r^2^	Korsmeyer–Peppas K_KP_ n		r^2^
FEP1, 1st stage	0.174 ± 0.0160	-	52.00 ^(b)^ ± 2.67	0.9995 ^(a)^ ± 0.0011	0.0160 ± 0.0013	0.9933 ^(b)^ ± 0.4200	0.0512 ± 0.0082	0.574 ± 0.029	0.9960 ± 0.3633
FEP1, 2nd stage	-	0.315 ± 0.022							
FEP2	-	0.680 ± 0.020	89.77 ± 4.09	0.9916 ± 0.0027	0.0180 ± 0.0009	0.9959 ^(c)^ ± 0.1360	0.0023 ± 0.0008	1.192 ± 0.061	0.9837 ± 0.0678
FEN3	0.0544 ± 0.0031	-	11.56 ± 0.54	0.9945 ± 0.0034	0.0437 ± 0.0045	0.9956 ^(d)^ ± 0.3719	0.1630 ± 0.016	0.472 ± 0.025	0.9408 ± 0.7519

k_0_—zero-order rate, k_1_—first-order rate, t_0.5_—half-release time, K_S_—Hixson–Crowell release rate, K_KP_—Kosmeyer–Peppas release rate, n—n factor in Korsmayer–Peppas model, r^2^—determination coefficient, ^(a)^—calculated for best fitted section of the data set, ^(b)^—calculated for entire data set, ^(c)^—calculated for best fitted section of the data set, ^(d)^—calculated for entire data set.
